# Sacral orientation and Scheuermann’s kyphosis

**DOI:** 10.1186/s40064-016-1772-x

**Published:** 2016-02-20

**Authors:** Smadar Peleg, Gali Dar, Nili Steinberg, Youssef Masharawi, Israel Hershkovitz

**Affiliations:** Department of Anatomy and Anthropology, Sackler Faculty of Medicine, Tel Aviv University, 69978 Ramat Aviv, Tel Aviv, Israel; Department of Physical Therapy, Zefat Academic College, Jerusalem Street 11, P.O. Box 160, 13206 Zefat, Israel; Department of Physical Therapy, Faculty of Social Welfare and Health Studies, University of Haifa, Mount Carmel, 31905 Haifa, Israel; The Zinman College of Physical Education and Sports Sciences at the Wingate Institute, 42902 Netanya, Israel; Department of Physiotherapy, School of Health Professions, Tel Aviv University, 69978 Ramat Aviv, Tel Aviv, Israel; Physical Anthropology Laboratory, Cleveland Museum of Natural History, 1 Wade Oval Drive, University Circle, Cleveland, OH 44106-1767 USA

**Keywords:** Kyphosis, Lordosis, Pelvic incidence, Scheuermann’s kyphosis, Spinal alignment

## Abstract

To examine whether the association between spinal alignment and sacral anatomical orientation (SAO) can be detected in skeletal populations, by comparing SAO values in individuals with a typical SD to individuals with normal spinal alignment. 2025 skeletons were screened for Scheuermann’s disease. Scheuermann’s kyphosis was established by the presence of apophyseal abnormalities associated with more than 5° of anterior wedging in each of three adjacent vertebrae. SAO was measured as the angle created between the intersection of a line running parallel to the superior surface of the sacrum and a line running between the anterior superior iliac spine and the anterior–superior edge of the symphysis pubis (PUBIS). SAO was measured on 185 individuals with normal spines and 183 individuals with Scheuermann’s kyphosis. Out of 2025 skeletons, 183 (9 %) were diagnosed with Scheuermann’s kyphosis. The sacrum was significantly more horizontally oriented in individuals with Scheuermann’s kyphosis compared with the control (SAO: 44.44 ± 9.7° vs. 50 ± 9.9°, p < 0.001). Alteration in spinal biomechanics due to a horizontally orientated sacrum may be an important contributing factor for the development of Scheuermann’s kyphosis.

## Background

Scheuermann’s disease (SD) is identified as a structural hyperkyphosis (Lowe 1990; Lowe and Line 2007; Scheuermann 1977, 1920). Two patterns of SD have been described in the pediatric population: a typical (thoracic) more common pattern (1–8 % of the population, Scoles et al. 1991; Sørensen 1964) and an atypical (thoracolumbar) less common one (0.4–4 % of the population, Blumenthal et al. 1987; Jansen et al. 2006; Lowe and Line 2007; Wood et al. 2012).

Many etiologies have been suggested for Scheuermann’s kyphosis. Until the end of the previous century there were already more than 12 theories [summarized and criticized by Alexander (1977)], and the number continues to grow in the twenty-first century (Damborg et al. 2012; DiGiovanni et al. 1989; Le Huec et al. 2011a; b; Lowe 1999; Lowe and Line 2007; Nissinen 1995; Roussouly et al. 2005; Scoles et al. 1991; Tsirikos and Jain 2011). The main controversy is related to the “mechanical” explanation offered by Alexander (1977) (Farrell et al. 2012).

Many current human diseases are the outcome of architectural compromises made in various elements of the body during the acquisition of erect posture, Scheuermann’s disease included. Acquiring a vertically oriented spine to almost its full length (sacrum excluded) is a prerequisite for erect posture; however, tilted pelvis, dorsally oriented sacrum and profound structural modifications of the muscles supporting the spine are mandatory for efficient (economic) bipedal locomotion and the bearing of large-headed babies (Abitbol 1987; Le Huec et al. 2011a, b; Mitchell 1934; Tague 1992). Pelvic tilt impacts the under-pelvis level, i.e., the angle of coxo-femoral joints in upright posture, whereas sacral slope impacts the above-pelvis level, i.e., spinal curves (Boulay et al. 2006). Nevertheless, the different alignment requirements of the structures expose the lumbosacral region to extreme shearing forces, the pathological consequences of which (e.g., spondylolysis, spondylolysthesis) have been widely studied (e.g., Hanson et al. 2007; Inoue et al. 2002; Labelle et al. 2005; Le Huec et al. 2011a; Marty et al. 2002; Peleg et al. 2009). On a broader perspective, the lumbosacral angle (LSA) expresses a compromise between the demands made by erect posture on the one hand, and bipedal locomotion on the other. Any major deviation from this angle might considerably elevate the stress on different parts of the spine (Roussouly et al. 2005; Scoles et al. 1991; Sørensen 1964).

The association between spinal deformities and pelvic orientation has been examined in several studies. The rationale behind this association relies on the fact that pelvic incidence is correlated with acquisition of walking during infancy and childhood (41.53° ± 8.3 in infants vs. 51.44° ± 10.85 in adults; Mac-Thiong et al. 2011; Mangione et al. 1997). Therefore, failure, due to whatever cause, to achieve adequate pelvic (or sacral) position, will trigger a chain reaction that may ultimately end in spinal deformity, as stated by Roussouly and Pinheiro-Franco (2011): “The genuine shape of the spine is probably one of the main mechanical factors of degenerative evolution” (p. S609). The latter authors have demonstrated that the spino-pelvic organization is significantly associated with the shape of lumbar lordosis: the higher the pelvic incidence (PI), the deeper the lordotic curve.

The literature supplies several clinical examples of the association between spino-pelvic organization and spinal anomalies: thus Marty et al. (2002) reported a correlation between sacral anatomic parameters and the development of spondylolisthesis; Mac-Thiong et al. (2003) found a very high pelvic incidence in scoliotic patients (57.3°) compared to the standard for normal adolescents (47.4°; Mac-Thiong et al. 2011).

Lately, a series of studies (Jiang et al. 2014; Tyrakowski et al. 2014, 2015) provided direct information regarding the sagittal spinal balance in SD patients. Jiang et al. (2014) were the first to report lower PI in adolescents with SD compared to controls (32° ± 10.8 vs. 45° ± 10.8), while Tyrakowski et al. (2014) were the first to present spinopelvic alignment in skeletally mature patients (Risser sign = 5) and to distinguish between the typical Scheuermann’s thoracic kyphosis (STK) and the atypical Scheuermann’s thoracolumbar kyphosis (STLK). Tyrakowski et al. (2015) also reported a significant difference in PI values when comparing between immature and mature patients with SD, using the Risser sign as an indicator for skeletal maturity (36.7° ± 8.1 vs. 39.4° ± 8.9).These three important studies were carried out on living subjects using lateral radiographs.

Recently, the normal range for human sacral anatomical orientation (SAO) has been presented (Peleg et al. 2007a, b). It has been shown that the greater the deviation from the normal range, the greater the stress on certain parts of the spine. This, together with the fact that pelvic incidence, sacral slope, degree of lordosis and degree of kyphosis are highly correlated (Boulay et al. 2006) implies that SAO may be a significant factor in the development of certain spinal deformities (Peleg et al. 2007a, b). To our knowledge, no study on the association between SAO and spinal alignment was carried out on skeletal populations. Although the shortcomings of such a study are clear, it possesses several advantages, namely: only spinal mal-alignments that are expressed in the vertebrae are considered as such, measurements are more accurate, and there is no effect of body weight or muscle tension on SAO.

The aim of the current study is to examine whether the association between spinal alignment and SAO can be detected in skeletal populations as well, by comparing SAO values in individuals with a typical SD to individuals with normal spinal alignment.

## Methods

### Materials

Ethical approval to work with ancient human material was granted by the Cleveland Museum of Natural History.

2025 skeletons from the human portion of the Hamann–Todd Osteologic Collection (curated at the Cleveland Museum of Natural History; Cleveland, Ohio, USA) were examined for the presence of Scheuermann’s disease. This collection consists of the defleshed skeletons of individuals who died in Cleveland during the first half of the twentieth century (African Americans and European Americans).

### Spinal deformity group

**Fig. 1:**
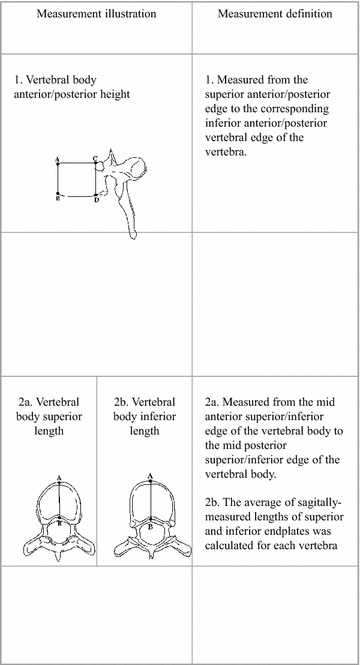
Vertebral body metric measurements

**Fig. 2:**
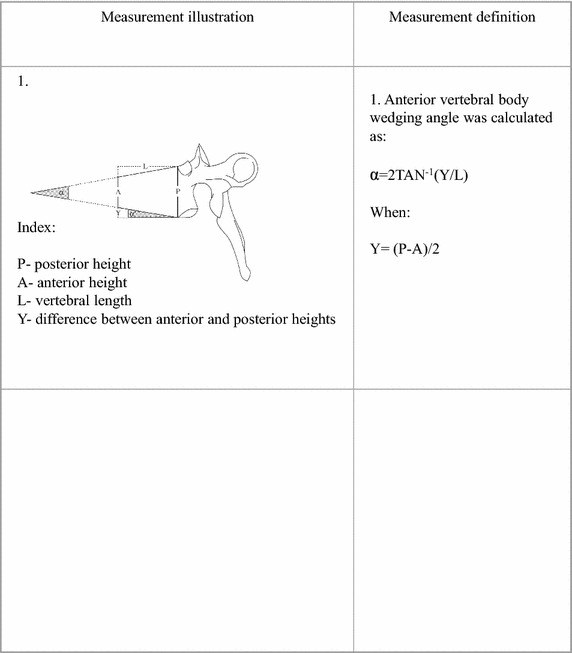
Vertebral body wedging angle

Scheuermann’s disease was established by the presence of apophyseal abnormalities associated with more than 5° of anterior wedging in each of 3 adjacent mid-thoracic vertebrae (T4–T9; Scoles et al. 1991; Sørensen 1964). Measurements were taken with a digital calliper to the accuracy of 0.1 mm. Thereafter, A-P (anteroposterior) angles were calculated (DiGiovanni et al. 1989; Figs. 1, 2).

### Control group

Age-matched control groups were constructed (from the non SD individuals) using a random number generator.

As positional registration of pelvic components was critical to this analysis, individuals with post-mortem pelvic deformations were excluded. Avoidance of confounding influences required exclusion of individuals with osteoporotic compression fractures. Specimens with sacralization of the fifth lumbar vertebra or lumbalization of the first sacral vertebra were also excluded.

### Sacral anatomical orientation

**Fig. 3:**
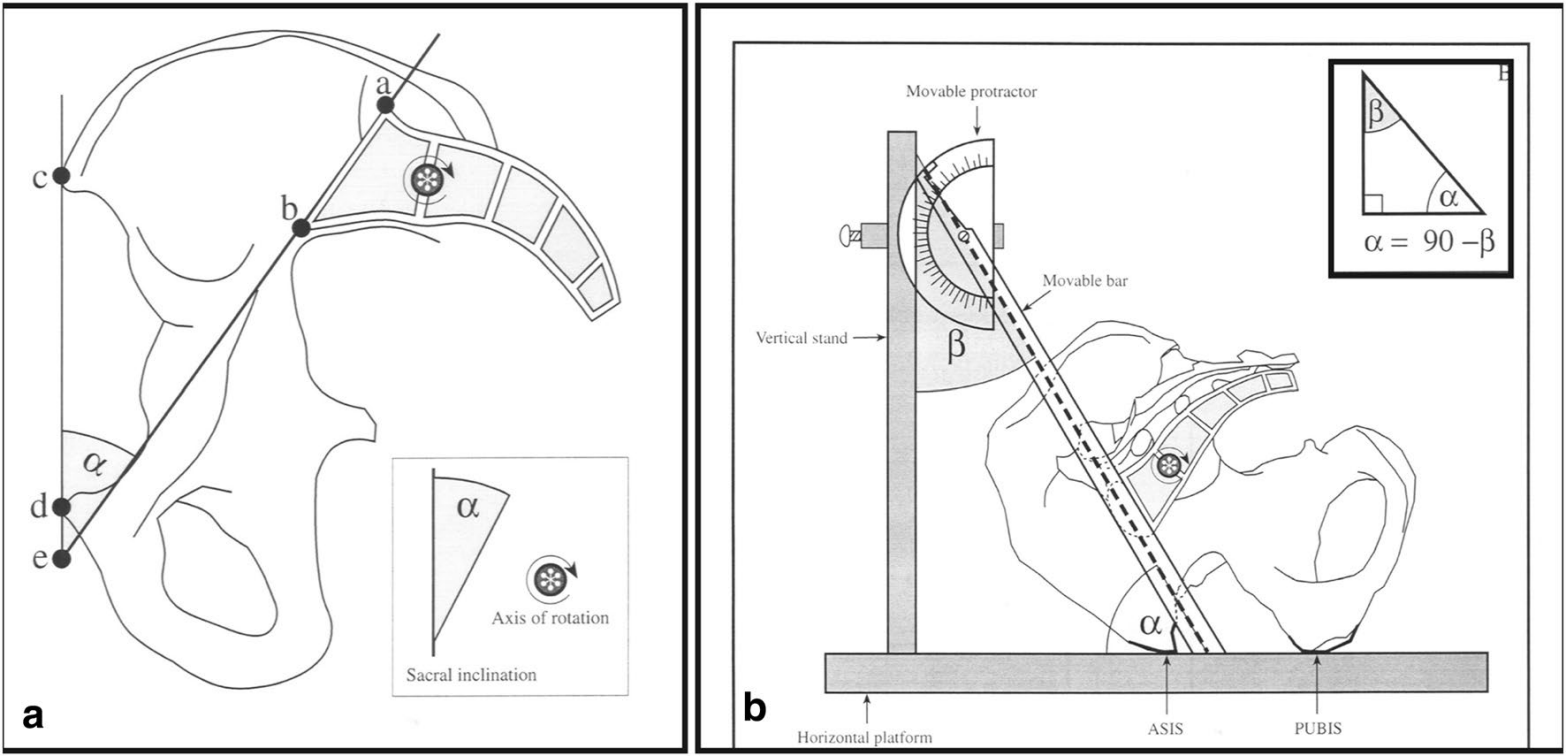
**a** Sacral anatomical orientation (SAO) definition (see text). **b** SAO measurement (see text)

Sacral anatomical orientation (SAO) is an angle measured between the tangent to S1 vertebral endplate and a line traced from the anterior superior iliac spine (ASIS) to the anterior–posterior edge of the pubis (Fig. 3a; Peleg et al. 2007a, b).

We used a specially designed device (Fig. 3b). The pelvis was placed on the measurement surface (ASIS and symphysis pubis facing downward) and we measured the angle created between the intersection of a line running parallel to the superior surface of the sacrum and a line running between the anterior–superior iliac spine (ASIS) and the anterior–superior edge of the symphysis pubis (PUBIS) (angle α; Peleg et al. 2007a; b).

### Power analysis

For α = 0.01 and β = 0.9 the minimal sample size required is n = 48.

### Statistical analysis

Four way-ANOVA was used to examine the relationship between SAO and Scheuermann’s disease, sex and ethnic origin. To further investigate the differences between the groups (with and without Scheuermann’s disease**)**, an unpaired t test was conducted. Statistical significant was set at p < 0.05.

## Results

Of the 2025 skeletons examined, 183 were diagnosed with Scheuermann’s kyphosis (9 %). 185 normal skeletons, selected via random number generator, established the control group. The demographic characteristics of the studied populations appear in Table 1. In Table 2 we present the metrical characteristics of the vertebral bodies in the mid-thoracic region in individuals with and without Scheuermann’s kyphosis. Significant differences are found in all vertebrae except T4.

**Table 1:** The demographic characteristics of the studied populations by spinal deformity

Group	European American				African Americans			
	n	X	SD	Range	n	X	SD	Range
Control	95	46.5	16.2	21–93	90	43.3	16.9	20–89
Scheuermann’s kyphosis	102	48.65	12.8	25–80	81	41.8	14.9	20–87

**Table 2:** Mean values of vertebral body heights and lengths (cm) (T4–T9) in European American males in the studied sample

Variable	Vert	European American normal spines (n = 53)	European American Scheuermann’s disease (n = 59)	p value
Anterior vertebral body height	T4	1.88 ± 0.12	1.87 ± 0.15	0.604
	T5	1.96 ± 0.11	1.88 ± 0.17	0.011
	T6	1.98 ± 0.10	1.88 ± 0.15	*<0.001*
	T7	2.01 ± 0.13	1.84 ± 0.16	*<0.001*
	T8	2.05 ± 0.10	1.86 ± 0.18	*<0.001*
	T9	2.13 ± 0.12	1.95 ± 0.17	*<0.001*
Posterior vertebral body height	T4	1.97 ± 0.17	1.99 ± 0.12	0.569
	T5	2.01 ± 0.27	2.12 ± 0.14	*0.004*
	T6	2.08 ± 0.12	2.18 ± 0.13	*<0.001*
	T7	2.13 ± 0.17	2.21 ± 0.13	*<0.001*
	T8	2.15 ± 0.10	2.24 ± 0.13	*<0.001*
	T9	2.21 ± 0.10	2.28 ± 0.13	*<0.001*
Superior vertebral body length	T4	2.23 ± 0.15	2.29 ± 0.17	0.056
	T5	2.42 ± 0.15	2.53 ± 0.19	*0.002*
	T6	2.61 ± 0.16	2.72 ± 0.20	*<0.001*
	T7	2.78 ± 0.18	2.94 ± 0.22	*<0.001*
	T8	2.94 ± 0.19	3.15 ± 0.23	*<0.001*
	T9	3.05 ± 0.20	3.24 ± 0.27	*<0.001*
Inferior vertebral body length	T4	2.36 ± 0.16	2.45 ± 0.16	*0.003*
	T5	2.54 ± 0.18	2.64 ± 0.19	0.010
	T6	2.71 ± 0.16	2.86 ± 0.22	*<0.001*
	T7	2.90 ± 0.18	3.08 ± 0.21	*<0.001*
	T8	3.02 ± 0.19	3.23 ± 0.28	*<0.001*
	T9	3.09 ± 0.20	3.27 ± 0.26	*<0.001*

Comment: significant difference (unpaired t test) after Bonferroni correction (0.005)

Index: vert, vertebral level

Significant differences are represented in italics

Multivariate analysis showed SAO to be sex and ethnicity independent. Therefore, in all further analyses all individuals were grouped together based on their spinal condition only: those manifesting Scheuermann’s kyphosis and those with normal spines.

Our statistical analysis showed that the sacrum was significantly more horizontally oriented in individuals with Scheuermann’s kyphosis compared with the control group: 44.44 ± 9.7° versus 50 ± 9.9° (p < 0.01).

## Discussion

In the present study, the sacrum was found to be significantly more horizontally oriented in the Scheuermann’s disease group compared to the control group. This finding is in line with other studies that have demonstrated significant association between spino-pelvic organization (e.g., sacral slope, pelvic incidence) and spine shape (Boulay et al. 2006; Le Huec et al. 2011a, b; Lowe 1990; Mac-Thiong et al. 2007, 2011; Mangione et al. 1997; Rose et al. 2009; Roussouly and Pinheiro-Franco 2011).

### Limitation of the study

This is a skeletal-based study and therefore sacral orientation may slightly differ in living individuals due to the action of the muscles and gravity. The SAO measurement is not intended to replace the PI measurement, but is offered as a useful tool in cases of skeletal material studies, or when the measurement of the hip axis is not possible.

**Fig. 4:**
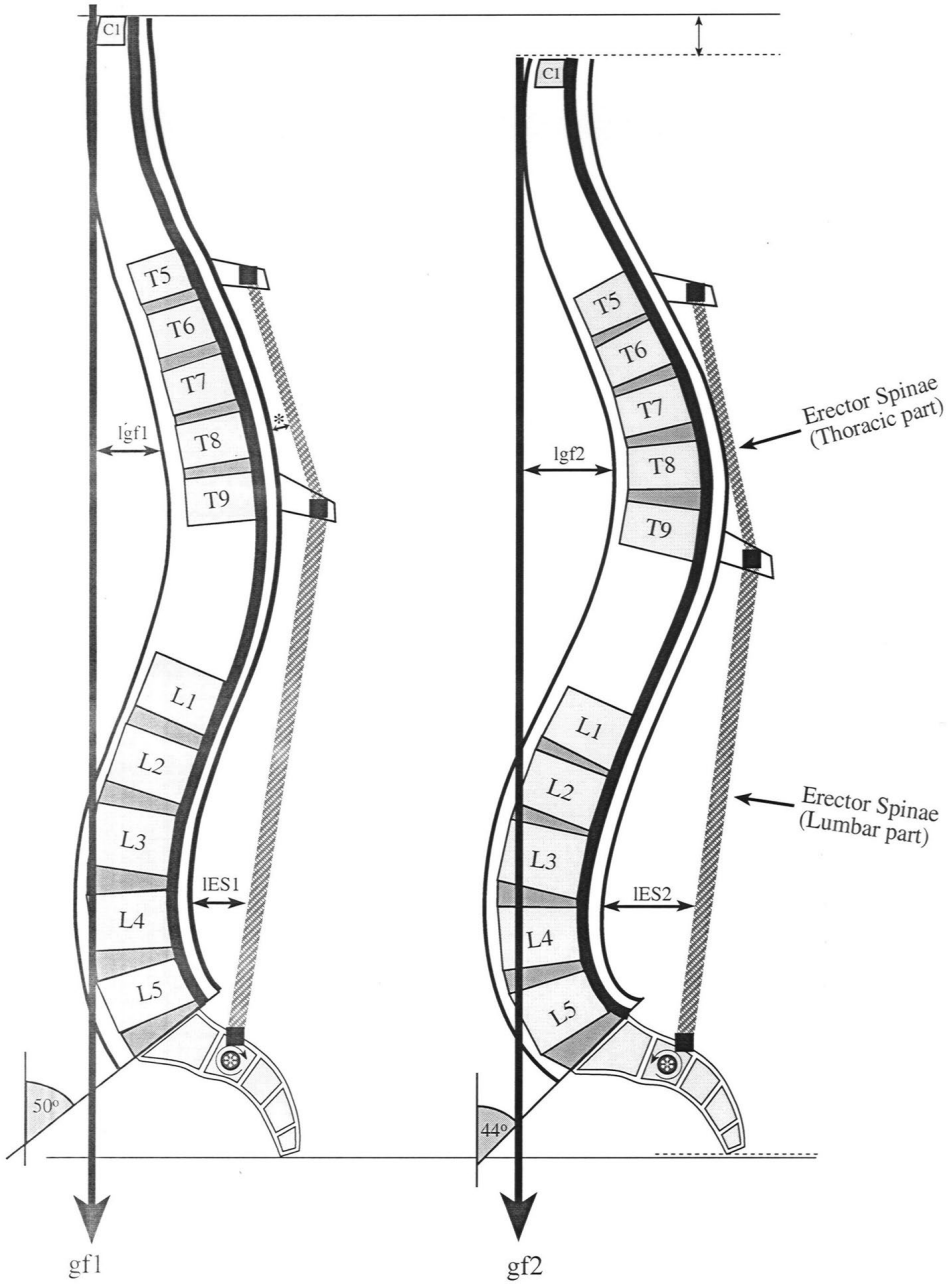
Kyphosis deformity mechanism—“Mechanical explanation”. Normal (*left*) versus deformed (*right*) spines. Note how the more horizontally oriented sacrum (*right*) alters the general shape of the spine and the distribution of force (Index: lg1, lever of gravity force in normal spines; lg2, lever of gravity force in deformed spines; lm1, lever of lumbar part of erector spine muscle in normal range of sacral inclination; lm2, lever of lumbar part of erector spine muscle in a decreased sacral inclination; gf1, gf2, gravity force in normal (1) versus deformed (2) spines)

Although this is not a longitudinal study and therefore a cause-effect relationship between sacral orientation and Scheuermann’s kyphosis is difficult to determine, we here propose a plausible explanation for this association:

Our major premise is that spinal curvatures must adapt to the orientation of the body of the sacrum. This is due to the fact that a horizontally oriented sacrum implies an almost vertical orientation of S1 superior discal surface. In order to cope with such a severe orientation of the discal surface, i.e., to maintain the integrity of the annular fibers of the L5–S1 intervertebral disc, lumbar lordosis must increase if a vertical spine is to be maintained. Hence, the more horizontally oriented the sacrum, the deeper the lordosis. This situation results in an increase in the lever of the lumbar extensors (Fig. 4). Therefore, in order to maintain the centre of gravity within the base of support (Fig. 4) in individuals with horizontally oriented sacrum, compensation in thoracic kyphosis must follow (i.e., increase in its concavity). The outcome of these complex relationships is increased pressure on the anterior segment of the mid thoracic vertebral bodies.

According to the Hueter–Volkmann law, increased pressure on the end plate of a bone inhibits its growth and conversely, reduced pressure accelerates growth (Stokes 2007; Stokes and Windisch 2006). Due to the above postural changes following a horizontally oriented sacrum, the lever arm of the line of gravity acting on the mid thoracic area towards flexion is increased, while the lever arm of the thoracic extensors decreases (Fig. 4). In the apical region, the attachments of the fibers of the longissimus and iliocostalis muscles are positioned too close to the axis of rotation, thereby greatly reducing their capacity of counterbalancing the force of gravity (Fig. 4). Inevitably, the changes in the spine’s configuration lead to alteration in force trajectories and intensity applied to the vertebral bodies. This may result in a growth disparity within vertebral bodies and eventually lead to the development of a beveled vertebra (Stokes and Windisch 2006; Tulsi 1971) and in severe cases, to thoracic kyphosis.

Several findings reported in the literature lend further support to the above explanation: It has been shown that final segmental spinal alignment starts with increased lumbar lordosis between ages 13–15 accompanied by a more horizontally oriented sacrum (Cil et al. 2004). Roussouly and Pinheiro-Franco (2011) have demonstrated that in adults, spinal curve shapes are associated with PI grade, i.e., advanced lumbar lordosis (type 4) is associated with high PI (i.e., a more horizontal sacrum). These findings are crucial for two reasons: first, they emphasize the role of sacral orientation in establishing normal spinal configuration, and second, they suggest that if normal growth processes are interrupted at these ages (ca. 13–15), the vertebral body will not develop properly. Once the epiphyseal rings are fully ossified with the vertebral bodies (at ca. 14–16 years), no further changes can occur (Bick and Copel 1951). Pediatric studies have shown that during the adolescent growth spurt, anterior vertebral growth exceeds posterior growth, resulting in a decrease in thoracic kyphosis and an increase in lumbar lordosis (Cil et al. 2004; Mac-Thiong et al. 2003, 2007, 2011; Murray and Bulstrode 1996; Pasha et al. 2010). In spines with a more horizontally oriented sacrum, this pattern of growth is interrupted as the anterior part of the vertebral body fails to reach its potential height due to increased pressure on this region, resulting in an increased kyphosis. This explanation fits with Alexander’s (1977) suggestion that Scheuermann’s disease is correlated with a static load in the flexed position, which corresponds with chair sitting. In such a position, the force applied to the anterior section of the vertebral body is greater than normal. Indirect support for our notion can also be found in Roussouly and Pinheiro-Franco’s (2011) study, showing that the contact force (CF = the sum of the force of gravity and the muscle action) acting on the posterior elements of hyperlordotic spines (i.e., more horizontally oriented sacrum) may cause spondylolisthesis (Roussouly and Pinheiro-Franco 2011). Following the same line of argument, given the fact that the upper lordotic arch inverts with the kyphotic arch, we can assume a high CF on the mid thoracic vertebral bodies, resulting in hyperkyphosis. These and other findings, e.g., Mac-Thiong et al.’s (2007) proposition that a correlation exists between a plumb line running anterior to the hip axis and the development of spinal pathology, lend support to the “mechanical basis” theory for the development of spinal deformities (Roaf 1958, 1960; Roussouly et al. 2005; Scoles et al. 1991; Stokes 2007; Villemure and Stokes 2009; Wood et al. 2012).

In partial contrast to our results, Jiang et al. (2014) and Tyrakowski et al. (2014) reported on lower PI in adolescent patients with SD compared to adults with normal spinal alignment. This apparent contradiction may be explained by the fact that SAO values change during spinal growth and during life by 15°:60° under the age of 20 compared to 52° between ages 21–40, 48° between ages 41–60, and 45° above the age of 61 (Peleg et al. 2007a) whereas PI values measured in the same study show only a small change with age, i.e., 3° from adult (21–40) to old population (above 61). This implies that PI is a less sensitive measurement than SAO in locating angular changes in sacrum orientation (Peleg et al. 2007a). Moreover, Tyrakowski et al. (2015) did find a significant difference in PI values between immature and mature patients with SD (using the Risser sign as an indicator for skeletal maturity) 36.7° ± 8.1 versus 39.4° ± 8.9, which supports our results.

Summing up the above, we can suggest a relationship between spinal alignment and the orientation of the sacrum. It is our understanding that it is the orientation of the sacrum that helps dictate the shape of the spine and not vice versa.

## Conclusions

Alteration in spinal biomechanics due to a horizontally orientated sacrum may be an important contributing factor for the development of Scheuermann’s kyphosis (SK; Fig. 4).
